# EHMTI-0373. Adrenal suppression associated with greater occipital nerve and multiple cranial nerve blocks using triamcinolone

**DOI:** 10.1186/1129-2377-15-S1-I5

**Published:** 2014-09-18

**Authors:** B Hywel, N Silver

**Affiliations:** 1 Neurology, Walton Centre For Neurology and Neurosurgery, Liverpool, UK

## Introduction

Greater Occipital Nerve (GON) and Multiple Cranial Nerve (MCN) blocks using local anaesthetics and corticosteroids have been used to treat various headache syndromes including Trigeminal Autonomic Cephalgias (TAC). We report cases where low cortisol levels have been seen in patients with TAC treated with GON/MCN blocks that included triamcinolone.

## Aims

We report four cases of adrenal suppression in TAC patients treated with GON/MCN blocks.

## Methods

The cases were collected retrospectively from a specialist headache clinic. Pituitary function tests prior to GON/MCN blocks were undertaken as part of their routine work up for TAC. Cortisol levels were repeated in the patients reported here due to varied nonspecific medical complaints.

## Results

Our cases include a 25 year old female with TAC who reported weight loss and leg pain following three MCN blocks in three weeks. Cortisol levels were found to be low on two separate occasions.

Another case is a 33 year old female treated with MCN blocks for TAC who was found to have low cortisol; the patient’s cortisol also failed to respond adequately to synthetic ACTH.

We aim to present another two patients, under ongoing investigation, who have had low cortisol levels associated with GON/MCN blocks for TACs.

## Conclusions

Whilst adrenal suppression is a known side effect of steroid use, it has not been widely described in patients treated with GON/MCN blocks. These cases suggest that professionals using local nerve blocks containing steroids should be cautious of the potential for adrenal suppression, particularly when used on a repeated basis.

No conflict of interest.

